# Supplementary material for: A non-monotonic code for event probability in the human brain
Source: Nat Commun. 2025 Nov 27;16:10695. doi: 10.1038/s41467-025-65727-7 (PMC12660876; doi:10.1038/s41467-025-65727-7)
Supplement: Supplementary file 1 — Supplementary Information [file 41467_2025_65727_MOESM1_ESM.pdf]

Supplementary Information

# A non-monotonic code for event probability in the human brain

Cedric Foucault[1,2,*,§], Tiffany Bounmy[1,*], Sébastien Demortain[1], Bertrand Thirion[3], Evelyn Eger[1], Florent Meyniel[1,4,§]

1. Cognitive Neuroimaging Unit, NeuroSpin (INSERM-CEA), University of Paris-Saclay, 91191 Gif-sur-Yvette, France
2. Sorbonne University, Doctoral College, F-75005 Paris, France
3. Inria, CEA, University of Paris-Saclay, Palaiseau, France
4. GHU Paris, psychiatrie et neurosciences, Hôpital Sainte-Anne, Institut de neuromodulation, 75014 Paris, France

[*]: co-first authors, [§]: corresponding authors cedric.foucault@gmail.com and florent.meyniel@cea.fr.

## Supplementary Notes

### Supplementary Note 1

Equation to be demonstrated:

$$p(h_{t+1} \mid s_{1:t+1}) \propto p(s_{t+1} \mid h_{t+1}) \int p(h_{t+1} \mid h_t)\, p(h_t \mid s_{1:t})\, dh_t \quad [1]$$

Below is the mathematical proof of the formula given by equation [1].

$$p(h_{t+1} \mid s_{1:t+1}) = p(h_{t+1} \mid s_{t+1}, s_{1:t})$$

$$p(h_{t+1} \mid s_{1:t+1}) = p(h_{t+1}, s_{t+1}, s_{1:t}) / p(s_{t+1} \mid s_{1:t})$$

$$p(h_{t+1} \mid s_{1:t+1}) = p(h_{t+1}, s_{t+1} \mid s_{1:t})\, p(s_{1:t}) / p(s_{t+1} \mid s_{1:t})$$

$$p(h_{t+1} \mid s_{1:t+1}) = p(s_{t+1} \mid h_{t+1}, s_{1:t})\, p(h_{t+1} \mid s_{1:t})\, p(s_{1:t}) / p(s_{t+1} \mid s_{1:t})$$

Conditional independence property (1): $s_{t+1}$ is conditionally independent of $s_{1:t}$ given $h_{t+1}$, therefore:

$$p(h_{t+1} \mid s_{1:t+1}) = p(s_{t+1} \mid h_{t+1})\, p(h_{t+1} \mid s_{1:t})\, p(s_{1:t}) / p(s_{t+1} \mid s_{1:t}) \quad [2]$$

The first term of equation [2] is the same as that equation [1]. Let's expand the second term of equation [2] using the sum rule.

$$p(h_{t+1} \mid s_{1:t}) = \int p(h_{t+1}, h_t \mid s_{1:t})\, dh_t$$

$$p(h_{t+1} \mid s_{1:t}) = \int p(h_{t+1} \mid h_t, s_{1:t})\, p(h_t \mid s_{1:t})\, dh_t$$

Conditional independence property (2) : $h_{t+1}$ is conditionally independent of $s_{1:t}$ given $h_t$, therefore

$$p(h_{t+1} \mid s_{1:t}) = \int p(h_{t+1} \mid h_t)\, p(h_t \mid s_{1:t})\, dh_t \quad [3]$$

By combining equations [2] and [3] and omitting the normalization factor $p(s_{1:t}) / p(s_{t+1} \mid s_{1:t})$, we obtain equation [1] which was to be demonstrated.

## Supplementary Results

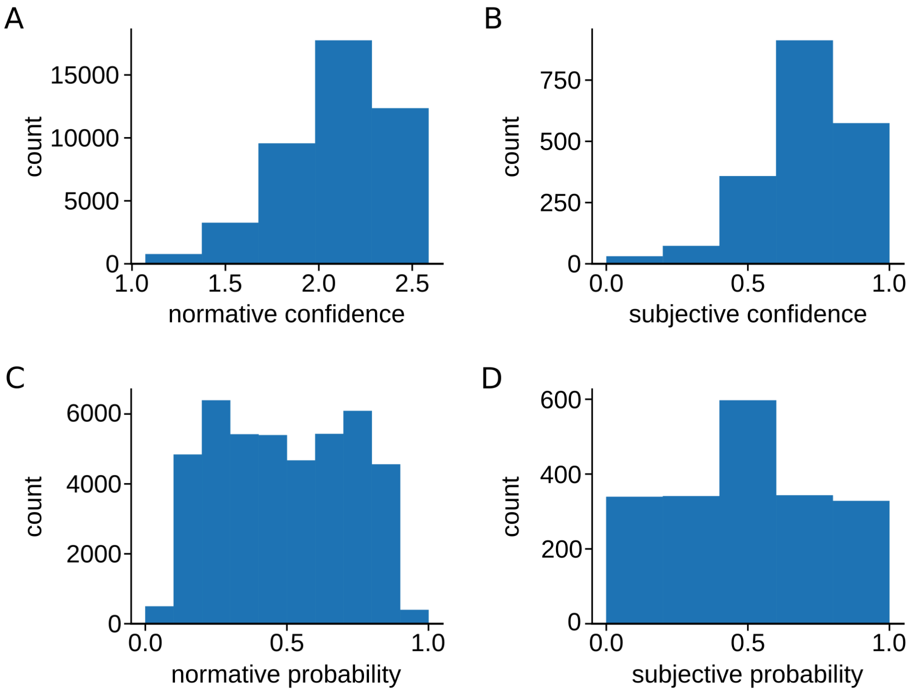


**Supplementary Figure 1. Distributions of probability and confidence in participants and the normative model.** (A, C) For the normative model (A, C), confidence and probability on all observations in all sequences are reported in the histogram. (B, D) For participants, reports were asked only occasionally (hence the lower counts). Note that the values of confidence differ between subjective reports and the model because the analog scale of subjective confidence has been mapped arbitrarily on the interval [0, 1], whereas optimal confidence is expressed in log precision units. For subjective probability, the central value (around 0.5) was reported more often than the others; note that it was also more often available. This is because the center of the middle bin is 0.5 for both the three-choice scale and the five-choice scale, whereas the center of the other bins differ across scales.

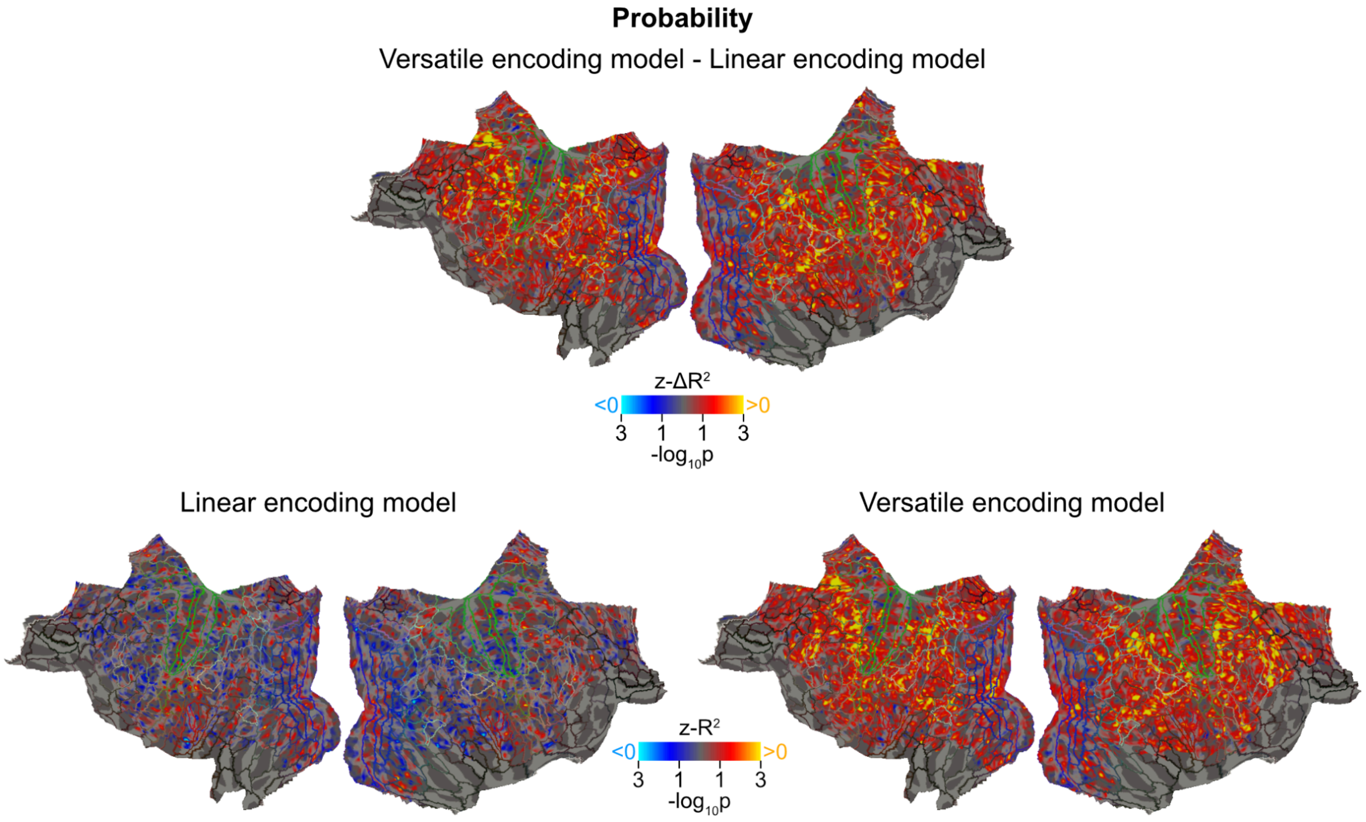


**Supplementary Figure 2. Difference in predictive accuracy between the versatile encoding model and the linear encoding model for probability.** Vertex-wise p-values are shown on flattened maps of the cortex. Top: P-values correspond to the group-level significance of z-$\Delta R^2$ scores obtained across participants ($\Delta R^2 = R^2$[versatile] - $R^2$[linear], cold/hot colors favor the linear/versatile encoding model respectively, p values are unthresholded and uncorrected). Bottom: Maps of the individual models, repeated from Fig. 4 for illustration purposes. Delineated by colored lines is the HCP-MMP1.0 parcellation [34].

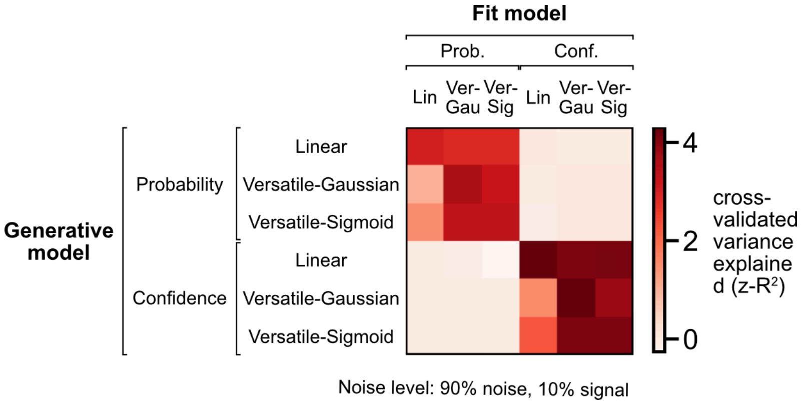


**Supplementary Figure 3. Simulation results with Gaussian and sigmoid basis functions.** Simulation results obtained as in Fig. 3B after splitting the versatile encoding model into two: one with Gaussian basis functions (the one used in the main text, referred to as Versatile-Gaussian above) and one with sigmoid basis functions (referred to as Versatile-Sigmoid above). The sigmoid basis functions of the Versatile-Sigmoid model are expressed $f_i(x) = 1 / [1 + exp[-k(x-\mu_i)]]$. For comparison, we took the same number of basis functions (10) and the same centers $\mu_i$ as for Versatile-Gaussian, and an equivalent slope $k$ was computed from the Versatile-Gaussian's $\sigma$ using the formula $k = 4 / [(2\pi)^{1/2}\sigma]$. In theory, radial basis sets have the same approximation properties in the limit of a large enough set, irrespective of the type of basis function used (e.g., Gaussian or sigmoid). In particular, Gaussian and sigmoid basis sets can approximate a nonlinear non-monotonic tuning curve. However, differences in the approximation properties can arise for small basis sets. The results presented in this figure indicate that here, both basis sets (with 10 basis functions) are roughly equivalent; if anything, the Gaussian basis set is better.

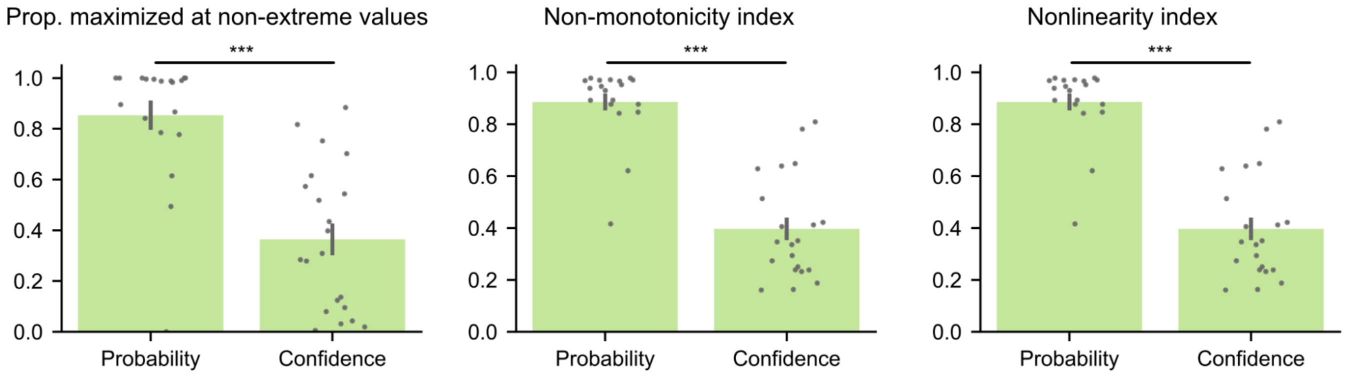


**Supplementary Figure 4. Results of the shape characterization without adding entropy and surprise as covariates during the estimation of tuning curves.** The analysis from Fig. 65C was repeated to characterize the tuning curves, but this time the weights for estimating the tuning curves were computed without including entropy and surprise as covariates in the encoding model. As shown above, the results are similar to those in Fig. 65C, and even show a greater difference between probability and confidence, with probability tuning curves becoming more non-monotonic nonlinear after removing the covariates. This confirms that the observed non-monotonic nonlinear profiles for probability, compared to confidence, do not arise as an artifact of including entropy and surprise as covariates.

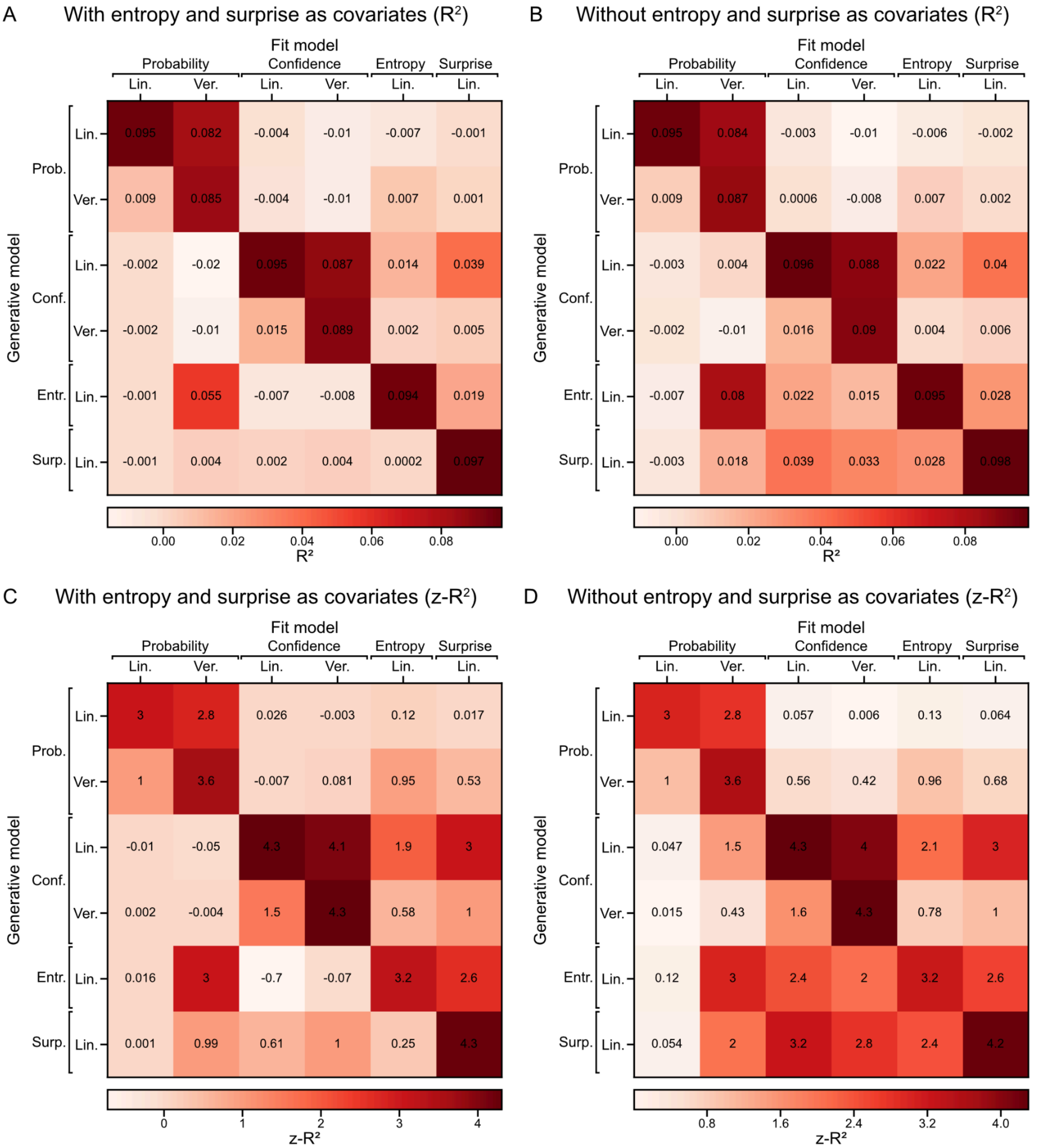


**Supplementary Figure 5. Comparison of simulation results when entropy and surprise are included (A and C) vs. not included (B and D) as covariates of the encoding model.** The simulation analysis from Fig. 32B was repeated considering two additional models, linear encoding of entropy and linear encoding of surprise, and in B and D, entropy and surprise were not included as covariates as done in the original analyses. As shown above, omitting these covariates leads to less separation between the codes, with higher scores for the probability and confidence encoding models when the generated activity actually encodes entropy or surprise.

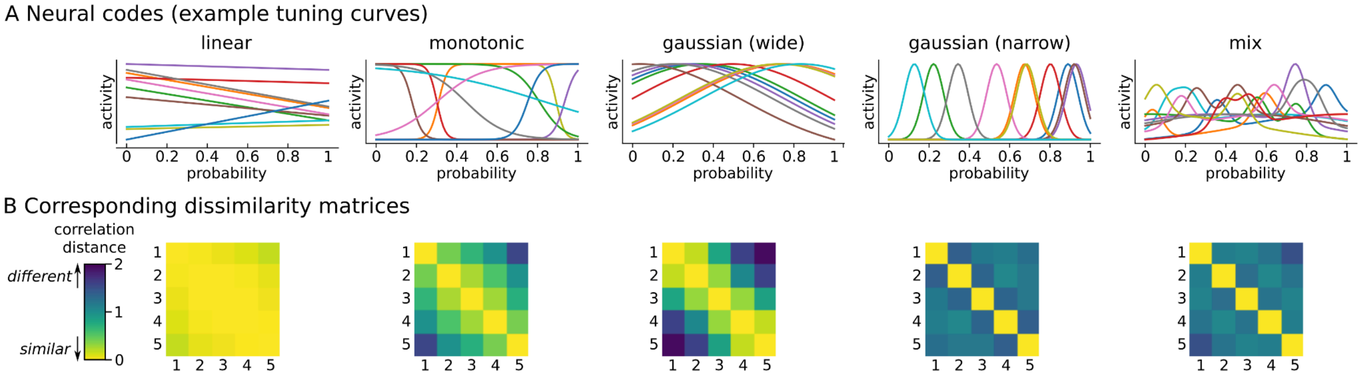


**Supplementary Figure 6. Simulated representational dissimilarity matrices for increasingly nonlinear codes.** (A) Five classes of code of probability with different degrees of nonlinearity were simulated. The degree of nonlinearity can be quantified with the characteristic measures shown in Fig. 65. In the linear code, the tuning curves are affine, with different slopes and intercepts across voxels; in the monotonic code, they are sigmoidal with different slopes and locations of the inflexion point; in the wide gaussian code, they are gaussians with SD=0.5 and different locations of the center; in the narrow gaussian code, the SD is 0.05; in the mix code, each tuning curve is the sum of one wide gaussian and two narrow gaussians. Nonlinearity increases from left (linear) to right (mix). Each simulation used 100 voxels, but only 10 are shown for visualization purposes, each with a different color. (B) Average representational dissimilarity matrices (RDMs) of patterns of voxel responses across five bins of probability, measured with correlation distance. The patterns of voxel responses were estimated using the versatile encoding model without cross-validation because these simulations are not corrupted by noise (and therefore, RDMs are symmetric). RDMs were computed for each simulated set of voxel responses, and averaged over 50 simulations. RDMs change from a graded matrix to the identity matrix as the code becomes more nonlinear (including, highly non-monotonic). Note that this property is illustrated here with the case of probability, but it is general and therefore applies to confidence.

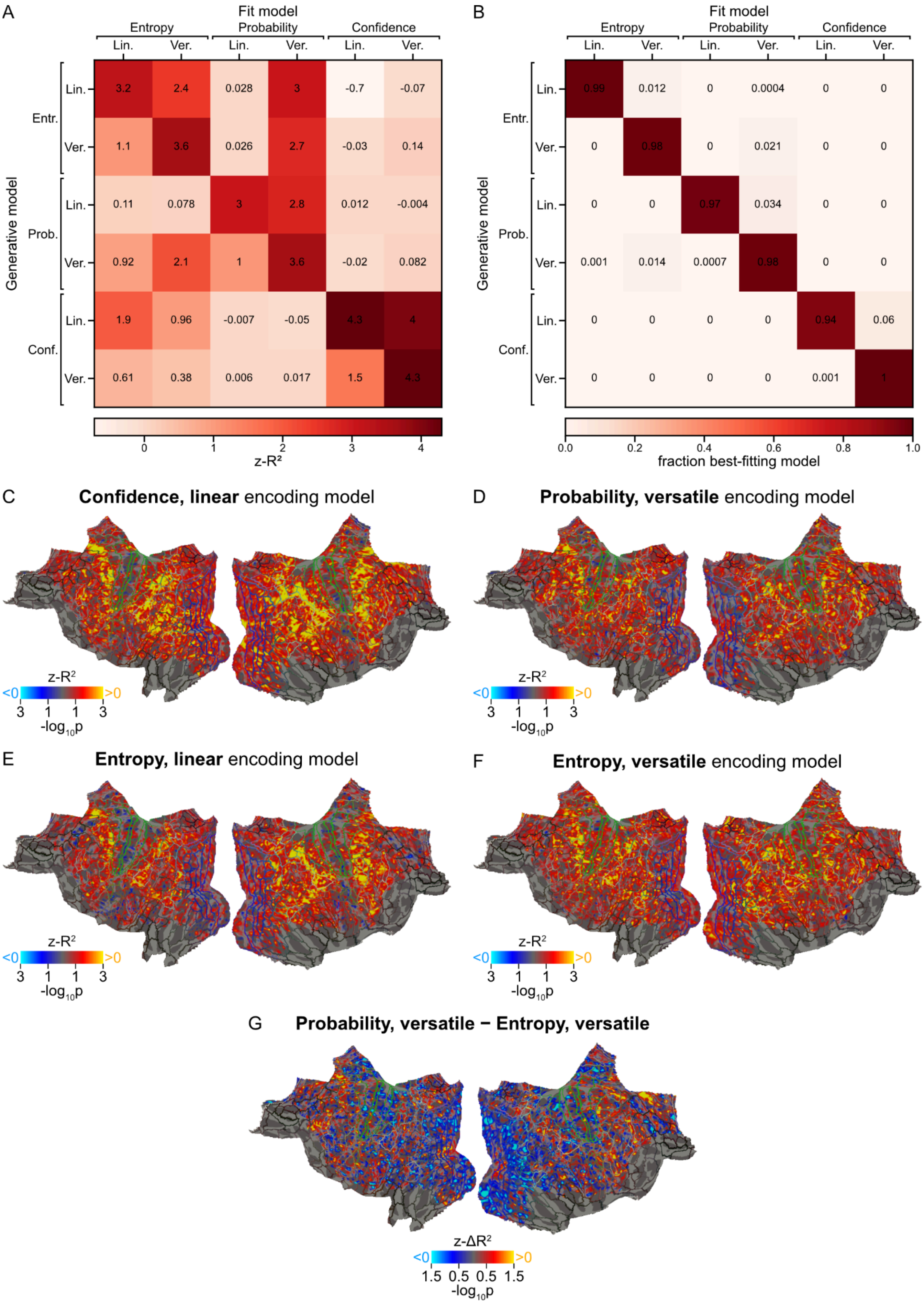
A
Fit model
Entropy
Probability
Confidence
Lin.
Ver.
Generative model
Entr.
Prob.
Conf.
3.2
2.4
0.028
3
-0.7
-0.07
1.1
3.6
0.026
2.7
-0.03
0.14
0.11
0.078
3
2.8
0.012
-0.004
0.92
2.1
1
3.6
-0.02
0.082
1.9
0.96
-0.007
-0.05
4.3
4
0.61
0.38
0.006
0.017
1.5
4.3
z-R²
B
0.99
0.012
0
0.0004
0.98
0.021
0.97
0.034
0.001
0.014
0.0007
0.98
0.94
0.06
0.001
1
fraction best-fitting model
C
Confidence, linear encoding model
D
Probability, versatile encoding model
E
Entropy, linear encoding model
F
Entropy, versatile encoding model
G
Probability, versatile − Entropy, versatile
z-R²
-log10p
z-ΔR²

**Supplementary Figure 7. The (linear) effect of confidence and the (non-monotonic) effect of probability are not confounded by a (linear) effect of entropy.** (A and B) Simulation results, repeating the analysis from Fig. 3B, including two additional models: a linear and a versatile encoding model of entropy. Entropy is a function of the probability estimate that quantifies the extent to which the next stimulus is unpredictable (maximum at p(A)=0.5 and gradually decreasing as p(A) deviates from 0.5). Plotting conventions are as in Fig. 3B. Note in (A) that some encoding models can partially explain data generated by another model (the linear entropy model partially explains data from a linear confidence model, the versatile entropy model partially explains data from a versatile probability model, and the versatile probability model partially explains data from linear and versatile entropy models). However, when models are directly compared, the model that best explains the data is almost always the generative model (panel B). (C–G) Cortical maps for the linear confidence model (C), versatile probability model (D), linear entropy model (E), versatile entropy model (F), and the difference in predictive accuracy between the versatile probability model and the versatile entropy model. Plotting conventions are as in Fig. 4. Panels C and D are reproduced from Fig. 5 and Fig. 4, respectively, to facilitate comparison with E and F. The linear effect of entropy seen in (E) largely overlaps with, but is less widespread than, the effect of confidence; it is thus likely arising from the small correlation between entropy and confidence. This effect is anatomically distinct from the (non-monotonic) effect of probability, which uniquely shows activity in the dorsolateral prefrontal cortex. Consistent with simulation results, the non-monotonic effect of entropy seen in (F) partly overlaps with the linear effect of entropy and the non-monotonic effect of probability. However, the comparison between the non-monotonic effects of probability and entropy in (G) shows a moderately stronger effect for probability in dorsolateral prefrontal regions.

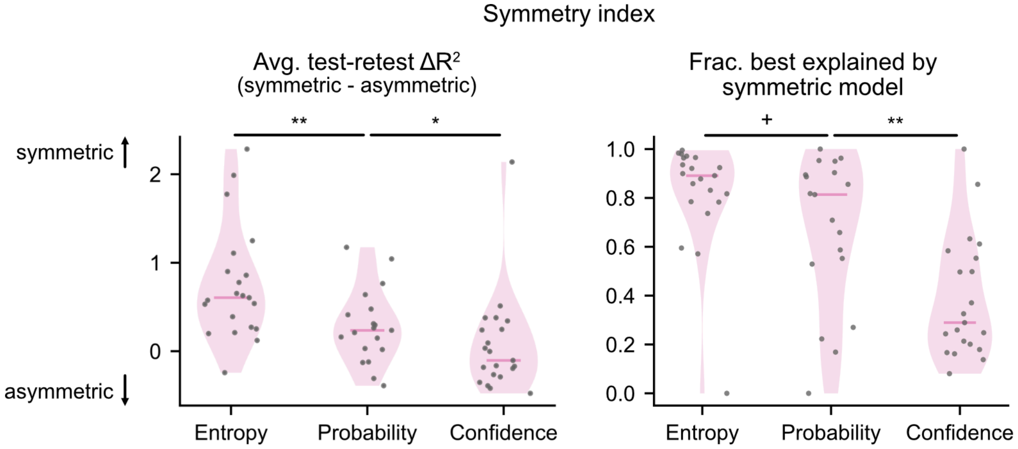


**Supplementary Figure 8. Symmetry index of the tuning curves in cortical vertices identified as encoding entropy, probability and confidence.** Entropy-coding vertices were identified using the same procedure as for probability and confidence, used in Figure 6, and tuning curves were estimated from the fMRI data in the same way. In vertices encoding entropy and probability, the tuning curves are those estimated using the versatile encoding model of probability; in vertices encoding confidence, the tuning curves are those estimated using the versatile encoding model of confidence. The tuning curves for entropy are expected to show a high degree of symmetry, since entropy is a symmetric function of probability (that is, symmetric with respect to the neutral probability value of 0.5). Symmetry was quantified by comparing two models for the tuning curves, a symmetric model and an asymmetric model, through a test-retest procedure where two independent halves of the data were used, one for fitting the models and one for testing the fitted models. In the symmetric model, the tuning curves were constrained to be symmetric; in the asymmetric model, no symmetry was imposed. The models were evaluated using the $R^2$, and the $R^2$ scores were compared between the two models. Two measures were computed as symmetry indices: the average difference in $R^2$ between the symmetric and asymmetric model (left plot) and the fraction of vertices best explained (according to the $R^2$) by the symmetric model (right plot). Statistical differences in the symmetry indices between entropy, probability and confidence were tested at the group level using a two-tailed Mann–Whitney U test. +: $p<0.1$; *: $p<0.05$; **: $p<0.005$.

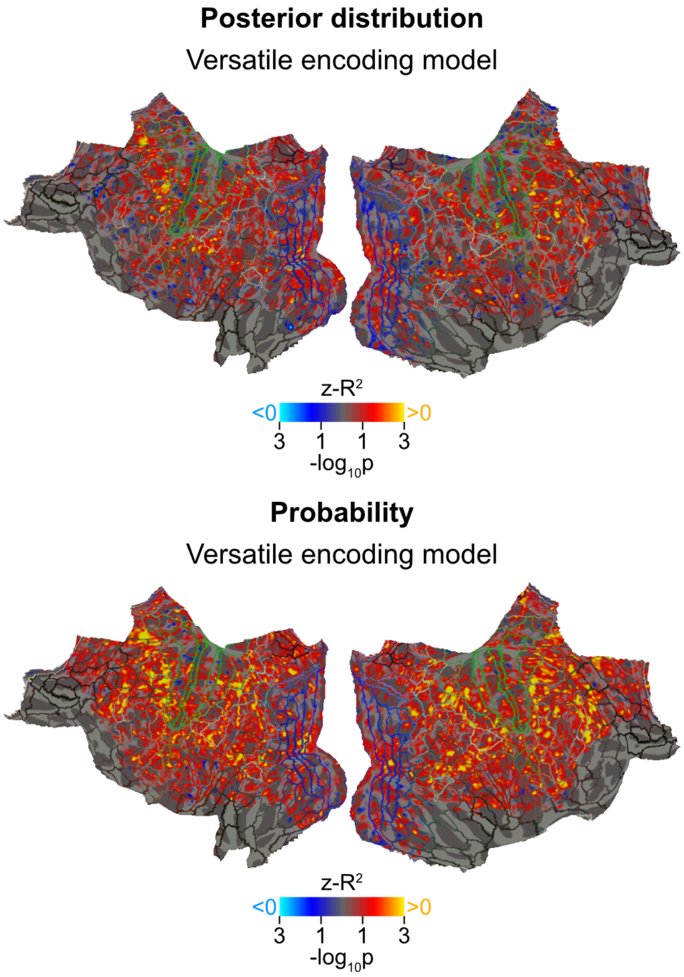


**Supplementary Figure 9. Predictive accuracy of the model encoding the posterior distribution (top) vs. the probability estimate (bottom)**. Cortical maps as in Fig. 43. See Methods for the encoding model of the posterior distribution. Bottom map is repeated from Fig. 43 for illustration purposes.

**Supplementary Table 1. Significant clusters explained by the linear encoding model for confidence.** The names of the cortical areas and parcels refer to the HCP-MMP1.0 atlas [34]. Peak x, y, z are MNI coordinates. Statistical analysis as in Fig. 4.

| Size (mm²) | Num. vertices | Hemisphere | Cortical area | Parcel | Peak x | Peak y | Peak z | Peak -log10(p) | Cluster $p_{FWE}$ |
|---|---|---|---|---|---|---|---|---|---|
| 1562.62 | 3701 | Right | Inferior_Parietal | IP2_R | 49.2 | -37.8 | 43 | 5.9237 | 0.0002 |
| 369.56 | 695 | Left | Paracentral_Lobular_and_Mid_Cingulate | SCEF_L | -9.4 | 4.5 | 56.4 | 5.7647 | 0.0002 |
| 341.32 | 676 | Right | Inferior_Parietal | PGi_R | 47.1 | -63.1 | 31.4 | 5.5385 | 0.0002 |
| 335.91 | 791 | Left | Superior_Parietal | 7PC_L | -36.9 | -50.4 | 54.9 | 6.1673 | 0.0002 |
| 257.7 | 601 | Left | Inferior_Parietal | PF_L | -49 | -38.4 | 43.1 | 6.115 | 0.0002 |
| 202.25 | 328 | Right | Premotor | 6r_R | 45.8 | 10.1 | 17.9 | 4.4931 | 0.0002 |
| 202.14 | 389 | Left | Premotor | FEF_L | -37 | -8.2 | 48.3 | 5.2117 | 0.0002 |
| 198.63 | 465 | Right | Premotor | 6a_R | 33.8 | -11 | 55.5 | 4.7601 | 0.0002 |
| 195.4 | 402 | Right | Paracentral_Lobular_and_Mid_Cingulate | SCEF_R | 7.7 | 5.8 | 51.2 | 4.6558 | 0.0002 |
| 193.77 | 432 | Right | Premotor | 6v_R | 56.1 | 6.7 | 25.7 | 5.6508 | 0.0002 |
| 189.66 | 567 | Right | Temporo-Parieto-Occipital_Junction | TPOJ1_R | 44.9 | -47.4 | 12.2 | 5.1703 | 0.0002 |
| 179.33 | 371 | Left | Temporo-Parieto-Occipital_Junction | STV_L | -55.6 | -48.2 | 13.3 | 6.1347 | 0.0002 |
| 175.3 | 458 | Left | Superior_Parietal | AIP_L | -30.9 | -38.3 | 41.6 | 5.2929 | 0.0002 |
| 171.23 | 209 | Right | Primary_Visual | V1_R | 17.8 | -94.6 | 0 | 4.1598 | 0.0002 |
| 164.69 | 241 | Right | Premotor | 6a_R | 30.6 | 1.4 | 49.2 | 4.9644 | 0.0002 |
| 155.42 | 293 | Left | Premotor | 6a_L | -29 | -2.3 | 45.8 | 5.92 | 0.0002 |
| 143.36 | 293 | Right | Paracentral_Lobular_and_Mid_Cingulate | 6ma_R | 16.3 | -0.9 | 66.3 | 4.8137 | 0.0002 |
| 142.98 | 326 | Right | Inferior_Parietal | PF_R | 48.4 | -37.2 | 26.8 | 4.2616 | 0.0002 |
| 137 | 362 | Right | Insular_and_Frontal_Opercular | FOP5_R | 32.5 | 27.2 | 7.9 | 6.2325 | 0.0002 |
| 134.03 | 253 | Left | Inferior_Parietal | PGs_L | -40.5 | -69.8 | 32.7 | 6.4739 | 0.0002 |
| 113.19 | 159 | Right | Early_Visual | V4_R | 26.5 | -81.6 | -8.8 | 4.9295 | 0.0002 |
| 110.53 | 228 | Left | Inferior_Parietal | PGi_L | -48.4 | -58.4 | 27 | 4.6878 | 0.0002 |
| 107.12 | 161 | Left | MT+_Complex_and_Neighboring_Visual_Areas | V3CD_L | -37.8 | -82.8 | 13.5 | 5.634 | 0.0002 |
| 105.53 | 192 | Left | Inferior_Parietal | PF_L | -55.1 | -35.6 | 28.7 | 5.1037 | 0.0002 |
| 95.02 | 201 | Right | Temporo-Parieto-Occipital_Junction | PSL_R | 58.3 | -43.3 | 22.7 | 4.4061 | 0.0002 |
| 89 | 195 | Left | Superior_Parietal | VIP_L | -19.5 | -64.9 | 56.8 | 4.2536 | 0.0002 |
| 88.28 | 175 | Right | Inferior_Frontal | IFJp_R | 33.6 | 5.6 | 31.4 | 3.9805 | 0.0002 |
| 86.02 | 135 | Left | Lateral_Temporal | PHT_L | -54.2 | -62.8 | 6.1 | 4.5631 | 0.0002 |
| 84.01 | 158 | Left | Premotor | 55b_L | -46.7 | 0.3 | 46.7 | 4.5385 | 0.0004 |
| 83.58 | 168 | Left | Inferior_Parietal | PGi_L | -43.4 | -67.9 | 19.6 | 5.5873 | 0.0004 |
| 81.09 | 113 | Right | MT+_Complex_and_Neighboring_Visual_Areas | MT_R | 42.5 | -73 | 0.7 | 3.9207 | 0.0006 |
| 79.95 | 128 | Right | Premotor | 55b_R | 42.9 | 2.3 | 43 | 4.7482 | 0.0006 |
| 75.19 | 89 | Left | Primary_Visual | V1_L | -15 | -100.9 | -4.8 | 4.2337 | 0.001 |
| 72.63 | 126 | Right | Dorsolateral_Prefrontal | 8C_R | 37 | 19.2 | 26.7 | 4.2202 | 0.0006 |
| 68.81 | 117 | Left | Dorsolateral_Prefrontal | p9-46v_L | -44.2 | 30.6 | 25.8 | 4.0961 | 0.0014 |

| | | | | | | | | | |
|---|---|---|---|---|---|---|---|---|---|
| 67.41 | 165 | Left | Inferior_Parietal | PFm_L | -48.4 | -49 | 43.7 | 4.6699 | 0.0016 |
| 61.37 | 150 | Left | Posterior_Opercular | 43_L | -57.8 | -0.7 | 14.5 | 4.6749 | 0.0024 |
| 55.58 | 83 | Left | MT+_Complex_and_Neighboring_Visual_Areas | MT_L | -44.6 | -72.1 | 6.3 | 4.5472 | 0.00499 |
| 54.71 | 112 | Left | Premotor | 6r_L | -46.6 | 2.4 | 26.3 | 3.6783 | 0.00559 |
| 54.6 | 71 | Left | Ventral_Stream_Visual | FFC_L | -41.7 | -64.7 | -18.7 | 4.9128 | 0.00579 |
| 53.38 | 92 | Left | Paracentral_Lobular_and_Mid_Cingulate | 6ma_L | -19.8 | -2.1 | 64.5 | 4.0547 | 0.00719 |
| 51.71 | 71 | Right | Early_Visual | V4_R | 25.9 | -71.9 | -5.4 | 5.8811 | 0.00898 |
| 50.52 | 115 | Right | Superior_Parietal | 7Pm_R | 8.8 | -64.1 | 48.9 | 4.279 | 0.01017 |
| 49.89 | 99 | Right | Superior_Parietal | 7PL_R | 13.3 | -71.6 | 52.2 | 4.3245 | 0.01057 |
| 48.04 | 123 | Left | Superior_Parietal | LIPd_L | -29.5 | -47.9 | 41 | 3.9552 | 0.01435 |
| 47.54 | 111 | Left | Inferior_Parietal | PGs_L | -40.1 | -63.9 | 33 | 4.5851 | 0.01534 |
| 46.66 | 81 | Left | Inferior_Parietal | IP1_L | -33 | -66.3 | 42 | 5.061 | 0.01713 |
| 46.55 | 87 | Right | Dorsolateral_Prefrontal | SFL_R | 6.6 | 2.9 | 65.2 | 3.8731 | 0.01772 |
| 45.58 | 70 | Left | MT+_Complex_and_Neighboring_Visual_Areas | V4t_L | -41.5 | -77.1 | -3.6 | 4.1141 | 0.01851 |
| 44.8 | 53 | Right | Dorsal_Stream_Visual | V7_R | 25 | -75 | 27.5 | 4.0285 | 0.02247 |
| 43.37 | 76 | Right | Anterior_Cingulate_and_Medial_Prefrontal | 8BM_R | 7 | 37.7 | 45.8 | 4.0643 | 0.02702 |
| 42.5 | 60 | Right | Inferior_Frontal | 44_R | 47.2 | 20 | 9.4 | 3.817 | 0.03056 |
| 41.13 | 62 | Right | Inferior_Parietal | PGp_R | 37.4 | -79.1 | 13.6 | 4.0701 | 0.03489 |
| 40.42 | 47 | Right | Dorsal_Stream_Visual | V7_R | 23.7 | -83.3 | 31.9 | 4.2199 | 0.03901 |
| 39.7 | 119 | Right | Posterior_Cingulate | PCV_R | 7.5 | -49.7 | 55.7 | 5.3128 | 0.04234 |
| 39.52 | 58 | Left | Inferior_Parietal | PGs_L | -35.1 | -75.9 | 39.4 | 3.7602 | 0.04273 |
| 39.51 | 104 | Left | Inferior_Parietal | PF_L | -59 | -31.3 | 36.7 | 3.9426 | 0.04273 |
| 38.84 | 95 | Left | Temporo-Parieto-Occipital_Junction | STV_L | -63.5 | -41.9 | 11.1 | 4.8528 | 0.04645 |

**Supplementary Table 2. Decoding accuracy for probability**. Names of cortical areas are as in Table 1. The decoding accuracy is compared to chance level (0.2) with a two-sided t-test. Only parcels with significant decoding accuracy ($p_{FDR}$<0.05) are reported.

| Cortical area | Parcel | Mean | T value | P value | $p_{FDR}$ |
|---|---|---|---|---|---|
| Dorsolateral_Prefrontal | 46 | 0.27 | 4.46 | 0.0002 | 0.0327 |
| Temporo-Parieto-Occipital_Junction | TPOJ2 | 0.27 | 4.03 | 0.0006 | 0.0327 |
| Inferior_Parietal | IP0 | 0.27 | 3.93 | 0.0007 | 0.0327 |
| Premotor | 6d | 0.28 | 3.84 | 0.0009 | 0.0327 |
| Posterior_Cingulate | 31pv | 0.27 | 3.76 | 0.0011 | 0.0327 |
| Superior_Parietal | AIP | 0.28 | 3.74 | 0.0012 | 0.0327 |
| Superior_Parietal | VIP | 0.28 | 3.70 | 0.0013 | 0.0327 |

**Supplementary Table 3. Decoding accuracy for confidence**. Names of cortical areas are as in Table 1. The decoding accuracy is compared to chance level (0.2) with a two-sided t-test. Only parcels with significant decoding accuracy ($p_{FDR}$<0.05) are reported.

| Cortical area | Parcel | Mean | T value | P value | $p_{FDR}$ |
|---|---|---|---|---|---|
| Premotor | 6a | 0.30 | 6.29 | 0.0000 | 0.0002 |
| MT+_Complex_and_Neighboring_Visual_Areas | PH | 0.34 | 6.18 | 0.0000 | 0.0002 |
| Inferior_Parietal | PFt | 0.34 | 5.87 | 0.0000 | 0.0003 |
| Superior_Parietal | VIP | 0.32 | 5.72 | 0.0000 | 0.0003 |
| Temporo-Parieto-Occipital_Junction | TPOJ1 | 0.33 | 5.59 | 0.0000 | 0.0004 |
| Inferior_Parietal | IP0 | 0.32 | 5.52 | 0.0000 | 0.0004 |
| Superior_Parietal | LIPd | 0.34 | 5.28 | 0.0000 | 0.0006 |
| MT+_Complex_and_Neighboring_Visual_Areas | V4t | 0.31 | 5.15 | 0.0000 | 0.0007 |
| Superior_Parietal | 7PC | 0.32 | 5.06 | 0.0000 | 0.0007 |
| Ventral_Stream_Visual | FFC | 0.31 | 5.04 | 0.0000 | 0.0007 |
| Premotor | 55b | 0.31 | 5.02 | 0.0000 | 0.0007 |
| Insular_and_Frontal_Opercular | FOP3 | 0.29 | 4.99 | 0.0000 | 0.0007 |
| Superior_Parietal | MIP | 0.32 | 4.81 | 0.0001 | 0.0010 |
| Temporo-Parieto-Occipital_Junction | STV | 0.31 | 4.75 | 0.0001 | 0.0010 |
| Dorsal_Stream_Visual | V7 | 0.30 | 4.75 | 0.0001 | 0.0010 |
| Early_Auditory | PFcm | 0.30 | 4.75 | 0.0001 | 0.0010 |
| MT+_Complex_and_Neighboring_Visual_Areas | V3CD | 0.30 | 4.66 | 0.0001 | 0.0012 |
| Inferior_Parietal | PGs | 0.33 | 4.64 | 0.0001 | 0.0012 |
| Paracentral_Lobular_and_Mid_Cingulate | 23c | 0.28 | 4.59 | 0.0001 | 0.0013 |
| Paracentral_Lobular_and_Mid_Cingulate | 5mv | 0.28 | 4.52 | 0.0002 | 0.0014 |
| Dorsal_Stream_Visual | IPS1 | 0.33 | 4.52 | 0.0002 | 0.0014 |
| Early_Visual | V3 | 0.31 | 4.42 | 0.0002 | 0.0016 |
| Posterior_Cingulate | POS2 | 0.29 | 4.40 | 0.0002 | 0.0016 |
| Lateral_Temporal | PHT | 0.30 | 4.40 | 0.0002 | 0.0016 |
| Posterior_Cingulate | PCV | 0.30 | 4.35 | 0.0002 | 0.0018 |
| Paracentral_Lobular_and_Mid_Cingulate | 6mp | 0.31 | 4.34 | 0.0003 | 0.0018 |
| Early_Visual | V4 | 0.30 | 4.32 | 0.0003 | 0.0018 |
| Somatosensory_and_Motor | 4 | 0.28 | 4.31 | 0.0003 | 0.0018 |
| Anterior_Cingulate_and_Medial_Prefrontal | p32pr | 0.31 | 4.29 | 0.0003 | 0.0018 |
| Inferior_Parietal | PFop | 0.29 | 4.28 | 0.0003 | 0.0018 |
| Paracentral_Lobular_and_Mid_Cingulate | 24dd | 0.28 | 4.26 | 0.0003 | 0.0018 |
| Premotor | 6d | 0.32 | 4.21 | 0.0004 | 0.0019 |
| Ventral_Stream_Visual | VMV2 | 0.28 | 4.21 | 0.0004 | 0.0019 |
| Early_Visual | V2 | 0.30 | 4.20 | 0.0004 | 0.0019 |
| Posterior_Opercular | FOP1 | 0.27 | 4.18 | 0.0004 | 0.0020 |
| Ventral_Stream_Visual | VVC | 0.30 | 4.12 | 0.0004 | 0.0022 |
| Ventral_Stream_Visual | PIT | 0.29 | 4.07 | 0.0005 | 0.0024 |
| Superior_Parietal | AIP | 0.30 | 4.00 | 0.0006 | 0.0028 |
| Auditory_Association | STSvp | 0.28 | 3.96 | 0.0007 | 0.0031 |
| Inferior_Parietal | PF | 0.29 | 3.89 | 0.0008 | 0.0035 |
| Premotor | FEF | 0.30 | 3.89 | 0.0008 | 0.0035 |
| Early_Auditory | RI | 0.30 | 3.88 | 0.0008 | 0.0035 |
| Somatosensory_and_Motor | 2 | 0.28 | 3.83 | 0.0009 | 0.0038 |
| Paracentral_Lobular_and_Mid_Cingulate | 6ma | 0.28 | 3.78 | 0.0011 | 0.0043 |
| Paracentral_Lobular_and_Mid_Cingulate | 5m | 0.29 | 3.77 | 0.0011 | 0.0043 |
| Dorsolateral_Prefrontal | 8Av | 0.27 | 3.75 | 0.0011 | 0.0044 |
| Insular_and_Frontal_Opercular | FOP4 | 0.30 | 3.74 | 0.0012 | 0.0044 |
| Temporo-Parieto_Occipital_Junction | PSL | 0.30 | 3.73 | 0.0012 | 0.0045 |
| Dorsolateral_Prefrontal | i6-8 | 0.27 | 3.70 | 0.0013 | 0.0047 |
| Inferior_Parietal | PGp | 0.28 | 3.69 | 0.0013 | 0.0048 |
| Superior_Parietal | LIPv | 0.30 | 3.67 | 0.0014 | 0.0048 |
| Temporo-Parieto-Occipital_Junction | TPOJ3 | 0.29 | 3.67 | 0.0014 | 0.0048 |
| Superior_Parietal | 7AL | 0.31 | 3.66 | 0.0014 | 0.0049 |
| Somatosensory_and_Motor | 1 | 0.29 | 3.64 | 0.0015 | 0.0050 |
| Superior_Parietal | 7Pl | 0.29 | 3.62 | 0.0016 | 0.0051 |

| | | | | | |
|---|---|---|---|---|---|
| Inferior_Parietal | IP1 | 0.29 | 3.55 | 0.0018 | 0.0058 |
| Dorsal_Stream_Visual | V3A | 0.28 | 3.55 | 0.0018 | 0.0058 |
| Auditory_Association | STSdp | 0.29 | 3.53 | 0.0020 | 0.0060 |
| MT+_Complex_and_Neighboring_Visual_Areas | FST | 0.29 | 3.51 | 0.0020 | 0.0060 |
| Inferior_Parietal | PFm | 0.27 | 3.51 | 0.0020 | 0.0060 |
| Early_Auditory | PBelt | 0.28 | 3.51 | 0.0020 | 0.0060 |
| Posterior_Cingulate | 7m | 0.29 | 3.44 | 0.0024 | 0.0070 |
| MT+_Complex_and_Neighboring_Visual_Areas | MST | 0.27 | 3.44 | 0.0024 | 0.0070 |
| Posterior_Cingulate | ProS | 0.27 | 3.42 | 0.0026 | 0.0072 |
| Dorsolateral_Prefrontal | 8C | 0.27 | 3.41 | 0.0026 | 0.0073 |
| MT+_Complex_and_Neighboring_Visual_Areas | LO1 | 0.28 | 3.39 | 0.0027 | 0.0074 |
| Inferior_Frontal | IFJa | 0.27 | 3.39 | 0.0027 | 0.0074 |
| Early_Auditory | A1 | 0.27 | 3.35 | 0.0030 | 0.0079 |
| Premotor | 6v | 0.27 | 3.32 | 0.0032 | 0.0084 |
| Posterior_Cingulate | DVT | 0.30 | 3.32 | 0.0033 | 0.0084 |
| Auditory_Association | A4 | 0.27 | 3.25 | 0.0038 | 0.0097 |
| Insular_and_Frontal_Opercular | MI | 0.26 | 3.25 | 0.0039 | 0.0097 |
| Somatosensory_and_Motor | 3b | 0.28 | 3.21 | 0.0042 | 0.0103 |
| Dorsal_Stream_Visual | V3B | 0.27 | 3.20 | 0.0043 | 0.0106 |
| Posterior_Opercular | OP4 | 0.27 | 3.19 | 0.0044 | 0.0107 |
| Inferior_Parietal | IP2 | 0.26 | 3.13 | 0.0051 | 0.0121 |
| Dorsolateral_Prefrontal | 9-46d | 0.27 | 3.08 | 0.0058 | 0.0136 |
| Paracentral_Lobular_and_Mid_Cingulate | SCEF | 0.28 | 3.05 | 0.0061 | 0.0141 |
| Premotor | 6r | 0.27 | 3.01 | 0.0068 | 0.0155 |
| Paracentral_Lobular_and_Mid_Cingulate | 5L | 0.27 | 2.99 | 0.0072 | 0.0160 |
| Primary_Visual | V1 | 0.28 | 2.98 | 0.0072 | 0.0160 |
| Superior_Parietal | 7Am | 0.27 | 2.95 | 0.0078 | 0.0169 |
| Paracentral_Lobular_and_Mid_Cingulate | 24dv | 0.26 | 2.95 | 0.0078 | 0.0169 |
| Ventral_Stream_Visual | V8 | 0.27 | 2.93 | 0.0081 | 0.0173 |
| Inferior_Frontal | IFJp | 0.28 | 2.93 | 0.0082 | 0.0174 |
| Temporo-Parieto-Occipital_Junction | TPOJ2 | 0.28 | 2.90 | 0.0088 | 0.0185 |
| Medial_Temporal | PHA1 | 0.27 | 2.87 | 0.0093 | 0.0193 |
| Inferior_Parietal | PGi | 0.27 | 2.86 | 0.0096 | 0.0196 |
| Early_Auditory | LBelt | 0.27 | 2.85 | 0.0098 | 0.0198 |
| MT+_Complex_and_Neighboring_Visual_Areas | MT | 0.28 | 2.81 | 0.0107 | 0.0215 |
| Posterior_Opercular | OP1 | 0.25 | 2.79 | 0.0114 | 0.0223 |
| Medial_Temporal | PreS | 0.27 | 2.78 | 0.0115 | 0.0223 |
| Inferior_Frontal | IFSa | 0.27 | 2.78 | 0.0115 | 0.0223 |
| Lateral_Temporal | TE2p | 0.26 | 2.75 | 0.0124 | 0.0237 |
| Insular_and_Frontal_Opercular | AVI | 0.26 | 2.74 | 0.0127 | 0.0240 |
| MT+_Complex_and_Neighboring_Visual_Areas | LO3 | 0.28 | 2.71 | 0.0135 | 0.0254 |
| Dorsal_Stream_Visual | V6A | 0.27 | 2.69 | 0.0142 | 0.0264 |
| Medial_Temporal | PHA2 | 0.27 | 2.62 | 0.0164 | 0.0301 |
| Ventral_Stream_Visual | VMV3 | 0.26 | 2.61 | 0.0168 | 0.0301 |
| Dorsolateral_Prefrontal | 46 | 0.26 | 2.61 | 0.0168 | 0.0301 |
| Auditory_Association | A5 | 0.27 | 2.61 | 0.0169 | 0.0301 |
| Ventral_Stream_Visual | VMV1 | 0.27 | 2.56 | 0.0190 | 0.0335 |
| Dorsal_Stream_Visual | V6 | 0.26 | 2.53 | 0.0202 | 0.0352 |
| Posterior_Cingulate | POS1 | 0.26 | 2.51 | 0.0210 | 0.0363 |
| Auditory_Association | STSda | 0.26 | 2.46 | 0.0237 | 0.0405 |
| Inferior_Frontal | IFSp | 0.25 | 2.45 | 0.0238 | 0.0405 |
| Insular_and_Frontal_Opercular | PoI2 | 0.26 | 2.45 | 0.0241 | 0.0405 |
| Somatosensory_and_Motor | 3a | 0.25 | 2.43 | 0.0250 | 0.0417 |
| Posterior_Cingulate | v23ab | 0.26 | 2.40 | 0.0266 | 0.0439 |
| Dorsolateral_Prefrontal | p9-46v | 0.25 | 2.40 | 0.0269 | 0.0441 |
| Posterior_Opercular | 43 | 0.25 | 2.37 | 0.0284 | 0.0461 |
| Anterior_Cingulate_and_Medial_Prefrontal | a24pr | 0.26 | 2.34 | 0.0304 | 0.0489 |
